# Vitamin D Increases Irisin Serum Levels and the Expression of Its Precursor in Skeletal Muscle

**DOI:** 10.3390/ijms24044129

**Published:** 2023-02-18

**Authors:** Lorenzo Sanesi, Manuela Dicarlo, Patrizia Pignataro, Roberta Zerlotin, Flavia Pugliese, Carla Columbu, Vincenzo Carnevale, Silvia Tunnera, Alfredo Scillitani, Maria Grano, Graziana Colaianni, Silvia Colucci

**Affiliations:** 1 Department of Translational Biomedicine and Neuroscience (DiBraiN), University of Bari Aldo Moro, Piazza Giulio Cesare 11, 70124 Bari, Italy; 2 Department of Precision and Regenerative Medicine and Ionian Area (DiMePRe-J), University of Bari Aldo Moro, Piazza Giulio Cesare 11, 70124 Bari, Italy; 3 Unit of Endocrinology, Fondazione IRCCS Casa Sollievo della Sofferenza, 71013 San Giovanni Rotondo, Italy; 4 Unit of Internal Medicine, Fondazione IRCCS Casa Sollievo della Sofferenza, 71013 San Giovanni Rotondo, Italy; 5 Department of Experimental and Clinical Medicine, University of Florence, Largo Brambilla 3, 50134 Firenze, Italy

**Keywords:** irisin, vitamin D, myoblast, hyperparathyroidism, Pgc1α, Sirt1

## Abstract

Irisin is a myokine synthesized by skeletal muscle, which performs key actions on whole-body metabolism. Previous studies have hypothesized a relationship between irisin and vitamin D, but the pathway has not been thoroughly investigated. The purpose of the study was to evaluate whether vitamin D supplementation affected irisin serum levels in a cohort of 19 postmenopausal women with primary hyperparathyroidism (PHPT) treated with cholecalciferol for six months. In parallel, to understand the possible link between vitamin D and irisin, we analyzed the expression of the irisin precursor, *Fndc5*, in the C2C12 myoblast cell line treated with a biologically active form of vitamin D, 1α,25-dihydroxyvitamin D3 (1,25(OH)2D3). Our results demonstrate that vitamin D supplementation resulted in a significant increase in irisin serum levels (*p* = 0.031) in PHPT patients. In vitro, we show that vitamin D treatment on myoblasts enhanced *Fndc5* mRNA after 48 h (*p* = 0.013), while it increased mRNAs of sirtuin 1 (*Sirt1*) (*p* = 0.041) and peroxisome proliferator-activated receptor γ coactivator 1α (*Pgc1α*) (*p* = 0.017) over a shorter time course. Overall, our data suggest that vitamin-D-induced modulation of Fndc5/irisin occurs through up-regulation of Sirt1, which together with Pgc1α, is an important regulator of numerous metabolic processes in skeletal muscle.

## 1. Introduction

Skeletal muscle acts similar to an endocrine organ by releasing molecules called myokines that are involved in regulating many of the positive effects of exercise on the body metabolism [1]. Irisin is a myokine that is produced during physical exercise both in humans and mice [2]. Physical activity leads to the production of peroxisome proliferator-activated receptor coactivator-1α (Pgc1α), which in turn promotes the expression of fibronectin type III domain-containing protein 5 (Fndc5), the precursor of irisin [2]. In humans, several studies have shown a positive association between circulating levels of irisin and bone mineral density [3,4]. Studies on murine models have documented the effect of irisin administration on increasing the bone mineral density (BMD), periosteal circumference, and polar moment of inertia in the long bones and in preventing bone loss and muscle wasting caused by musculoskeletal unloading [5,6].

Ongoing scientific research aims to identify the factors that influence irisin production in health and disease. In our previous studies, we found a negative association between irisin and vitamin D in a cohort of patients affected by Charcot–Marie–Tooth (CMT) disease [7] and in children and adolescents with type 1 diabetes mellitus (T1D) [8]. Interestingly, pediatric patients with Prader Willi syndrome (PWS) not supplemented with vitamin D showed lower irisin levels than both the controls and patients supplemented with vitamin D [9]. In a cohort of elderly patients with type 2 diabetes mellitus (T2D) and vitamin D deficiency, vitamin D supplementation increased the irisin serum levels, along with Sirt1 levels [10]. Sirt1 belongs to Sirtuins, a family of class III histone deacetylases, and plays a key role in the deacetylation and activity of Pgc1α/ERRα receptor complexes, which are essential for regulating the metabolism of transcription factors [11]. Sirt1 levels can be reduced in chronic diseases such as diabetes, thus an increase in its expression is crucial for improving glucose indices and mitochondria function [10].

Vitamin D is a molecule with a well-known function in musculoskeletal metabolism [12,13]. Considering the similar action of irisin as a key factor in bone and muscle health, and as some studies have uncovered an existing correlation between these two molecules, the rationale of our study was to investigate the interplay between irisin and vitamin D more thoroughly. Therefore, we measured irisin serum levels in a cohort of patients affected by primary hyperparathyroidism (PHPT) treated for 6 months with vitamin D. In PHPT, a pathological condition caused by an adenoma of the parathyroid, vitamin D insufficiency is more common than in the general population and, although its supplementation is still debated, lower vitamin D levels are associated with more severe disease [14,15,16,17].

The results presented herein show that vitamin D treatment in PHPT patients for six months increased the serum concentration of irisin compared with baseline. Given this clinical outcome, we used a cellular model to understand the mechanisms underlying the possible action of vitamin D on irisin synthesis, choosing the skeletal muscle cell as our in vitro study prototype, which is the main source of circulating irisin.

Therefore, we also performed an in vitro study analyzing the expression of the irisin precursor, *Fndc5*, in myoblasts treated with 1α,25-dihydroxyvitamin D3, and the possible pathways involved in this metabolic signaling.

## 2. Results

### 2.1. Patients and Data Collection

For the assessment of serum irisin levels in the present study, postmenopausal women were selected because PHPT is prevalent in this group of patients. In addition, because it is common to observe vitamin D deficiency in this condition, and considering that some of the PHTP-related damage, at least in part, could be attributable to vitamin D deficiency, we enrolled 16 Caucasian postmenopausal female patients, aged 58.69 ± 7.54 years, treated with Dibase (2000 U/day) at the diagnosis of PHPT until follow-up after 6 months. This subgroup of patients had not undergone parathyroidectomy, as the mean parathyroid hormone (PTH) values were significantly lower than in patients undergoing surgery. Table 1 shows the demographic, anthropometric, and laboratory parameters of patients enrolled for the assessment of serum irisin levels.

### 2.2. Vitamin D Supplementation Increases Irisin Serum Levels in PHPT Patients

**Figure 1:**
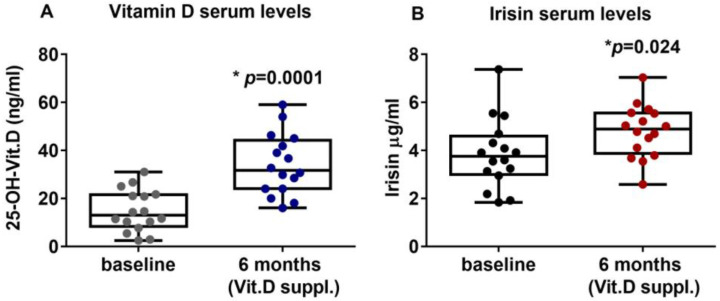
After six months of treatment with vitamin D (cholecalciferol), PHPT patients with hypovitaminosis D showed increased serum levels of 25-OH-Vitamin D (**A**) and irisin (**B**). Data are presented as box-and-whisker plots with median and interquartile ranges, from max to min, with all data points shown. Mann–Whitney test was used to compare groups. *p* value as indicated; * significant *p* value.

PHPT patients with hypovitaminosis D were treated for six months with vitamin D (Dibase, 2000 U/day). The results showed that the serum levels of vitamin D were significantly increased, thus suggesting that the supplementation effectively improved the hypovitaminosis condition, enhancing vitamin D levels from a mean value of 14.83 ± 8.66 ng/mL (95%CI 10.21 to 19.44) at the baseline to a post-treatment mean value of 34.1 ± 12.71 ng/mL (95%CI 27.33 to 40.87) (*p* = 0.0001) (Figure 1A). Interestingly, after six months of Vitamin D supplementation, irisin serum levels were significantly increased to 3.85 ± 1.44 μg/mL (95%CI 3.08 to 4.62) compared with 4.79 ± 1.09 μg/mL (95%CI 4.21 to 5.38) at baseline (*p* = 0.024) (Figure 1B). Interestingly, our results showed that PTH circulating levels after 6 months of vitamin D treatment (median (IQR): 106.7 (78.5–133.8) pg/mL) were slightly reduced compared with baseline (median (IQR): 116 (76.75–134.5) pg/mL), although not significantly (*p* = 0.79).

### 2.3. Vitamin D Increases Fndc5 Expression in Myoblasts

**Figure 2:**
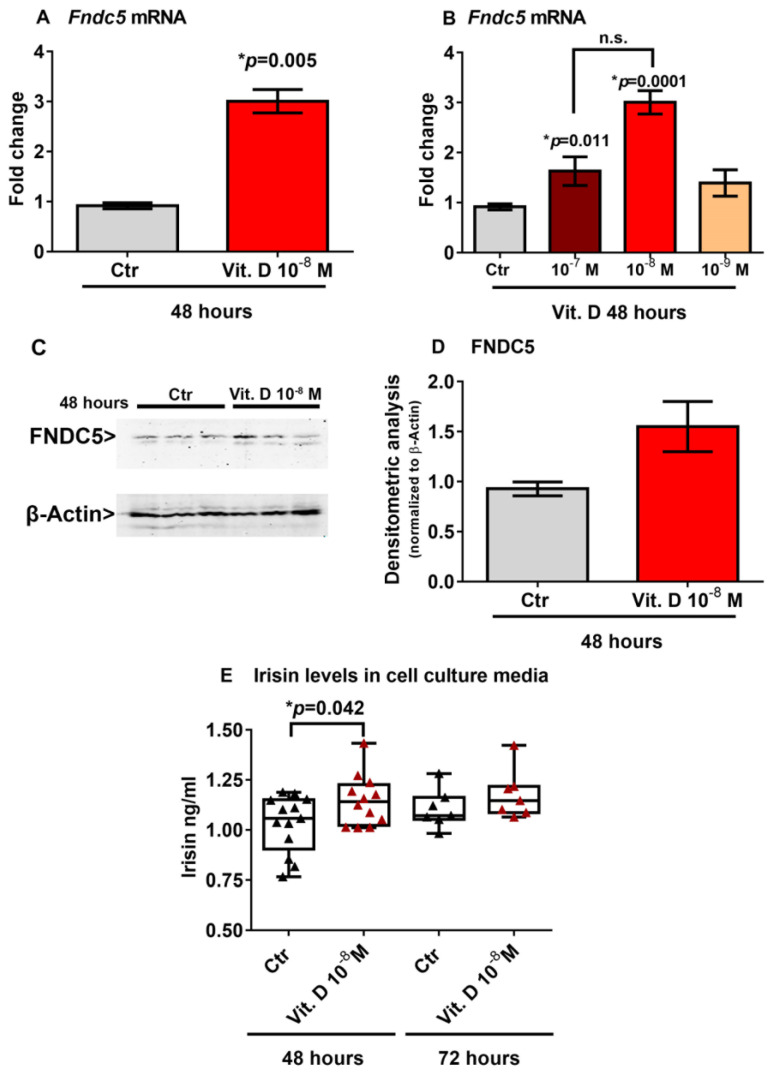
Treatment with vitamin D increases *Fndc5* mRNA expression after 48 h (**A**). The lowest effective dose of vitamin D for increasing *Fndc5* was 10^−8^ M (**B**). Western immunoblotting (**C**) and densitometric quantitation (**D**) of Fndc5 expression in myoblasts after 48 h of treatment with 10^−8^ M vitamin D, normalized to control loading (β-actin) (Western blot image of *n* = 3 Ctr and *n* = 3 vitamin D treated samples). Data are presented as mean ± SEM. Unpaired *t*-test (**A**–**D**) and ANOVA (**B**) were used to compare the groups. Levels of irisin measured by ELISA assay in cell culture media from myoblasts treated for 48 and 72 h with 10^−8^ M vitamin D (**E**). Data are presented as box-and-whisker plots with median and interquartile ranges, from max to min, with all data points shown. The Kruskal–Wallis test is used to compare groups. *p* value as indicated; * significant *p* value; n.s. not significant.

To investigate a possible effect of vitamin D on *Fndc5* mRNA expression, we stimulated myoblasts with 1α,25-dihydroxyvitamin D3 (vitamin D). We found that 10^−8^ M vitamin D increased the *Fndc5* mRNA expression after 48 h (Figure 2A). Considering the physiological range of 30–40 ng/mL concentration of vitamin D in human serum, corresponding to 7.5 × 10^−8^ M and 1 × 10^−7^ M, we also treated myoblasts with a higher (10^−7^ M) and lower (10^−9^ M) dose. Of note, the dose–response experiment showed that both 10^−7^ M and 10^−8^ M were effective at increasing *Fndc5* mRNA, although the latter dose to a greater extent (Figure 2B). To further validate the vitamin-D-mediated increase on *Fndc5* expression in myoblasts, we measured its protein levels. Western blot analysis showed that treatment with 10^−8^ M vitamin D for 48 h slightly increased the Fndc5 expression (Figure 2C,D). Moreover, the irisin concentration released in the cell culture media of vitamin-D-treated myoblasts was significantly higher than the control cells (Figure 2E). We detected a significantly higher amount of secreted protein in vitamin-D-treated cell culture media compared with those of the control cells, but not the same increase in Fndc5 expression in cell lysates, probably due to the rapid cleavage in the irisin precursor, whose expression thus appeared to be only slightly increased in myoblasts. Additionally, the analysis of the cell culture media after 72 h of vitamin D treatment still showed a slight upward trend, although not significant.

### 2.4. Vitamin D Increase the Expression of Sirt 1 and Pgc1α

**Figure 3:**
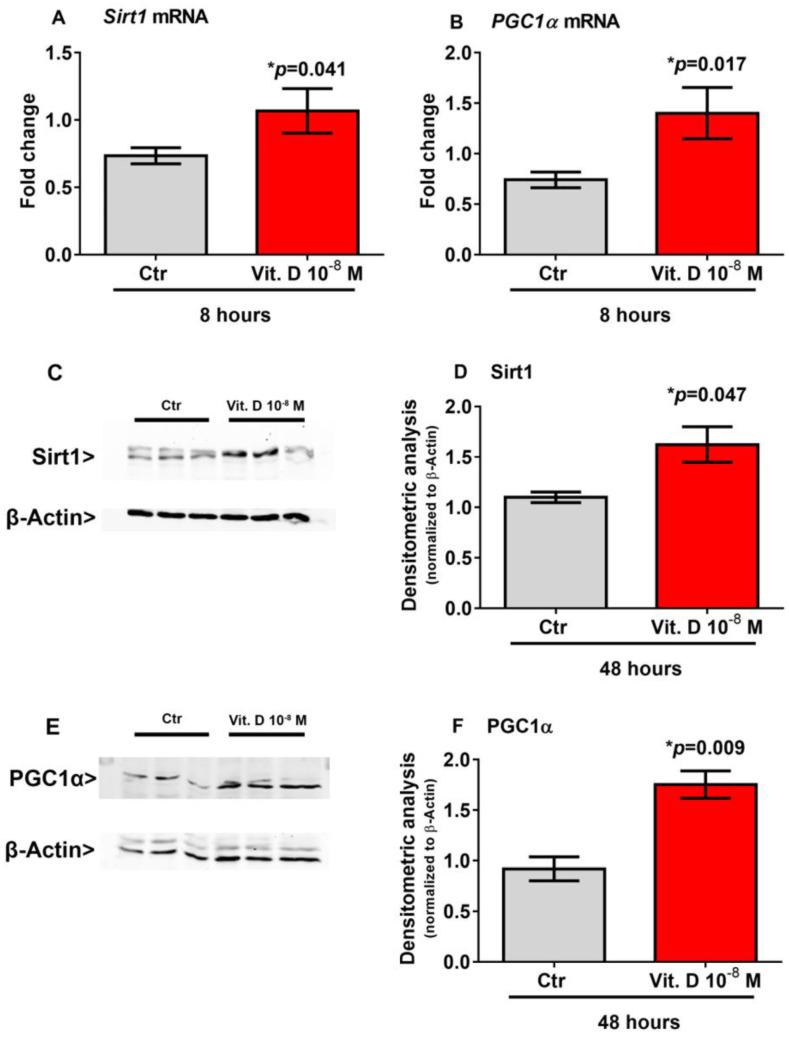
Eight hour treatment with 10^−8^ M 1α,25-dihydroxyvitamin D3 (vitamin D) increases *Sirt1* (**A**) and *Pgc1α* (**B**) mRNA expressions. Western immunoblotting (**C**) and densitometric quantitation (**D**) of Sirt1 protein expression in myoblasts after 8 h of treatment with 10^−8^ M vitamin D, normalized to control loading (β-actin) (Western blot image of *n* = 3 Ctr and *n* = 3 vitamin D treated samples). Western immunoblotting (**E**) and densitometric quantitation (**F**) of Pgc1α protein expression in myoblasts after 8 h of treatment with 10^−8^ M vitamin D, normalized to control loading (β-actin) (Western blot image of *n* = 3 Ctr and *n* = 3 vitamin D treated samples). Data are presented as mean ± SEM. The unpaired *t*-test is used to compare the groups. *p* value as indicated; * significant *p* value.

Recent evidence has shown that Sirt1 increases the transcription of the *Pgc1α* gene [18,19], which in turn is the master regulator of *Fndc5* gene expression [2]. Therefore, to explore possible mediators involved in the vitamin D/irisin pathway, we treated myoblasts with vitamin D for a shorter time-course than that required to induce the upregulation of *Fndc5*. Our results showed that 8-h stimulation with vitamin D significantly increased the expression of *Sirt1* mRNA (*p* = 0.041) (Figure 3A) and its protein level (*p* = 0.047) (Figure 3C,D) and the expression of *Pgc1α* mRNA (*p* = 0.017) (Figure 3B) and its protein level (*p* = 0.009) (Figure 3E,F) in skeletal muscle cells.

### 2.5. Sirt1 Is Involved in Vitamin-D-Mediated Upregulation of Fndc5

**Figure 4:**
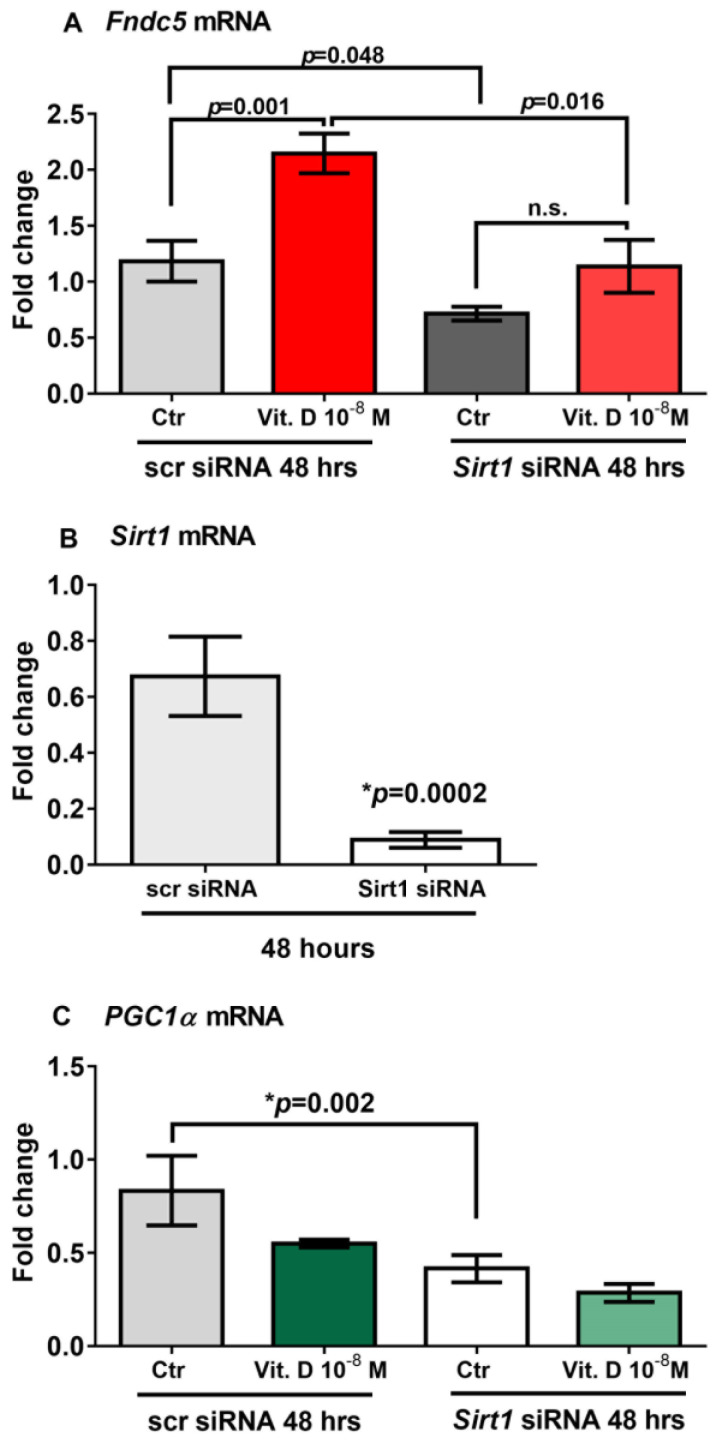
Forty-eight hours of treatment with 10^−8^ M 1α,25-dihydroxyvitamin D3 (vitamin D) fails to increase *Fndc5* mRNA in *Sirt1*-silenced myoblasts (Sirt1 siRNA) (**A**). *Sirt1* mRNA expressions in *Sirt1*-silenced myoblasts for 48 h (*Sirt1* siRNA) (**B**). In the absence of *Sirt1*, *Pgc1α* mRNA expression is significantly reduced (**C**). Data are presented as mean ± SEM. Unpaired *t*-test (**B**) and ANOVA (**A**–**C**) are used to compare groups. *p* value as indicated; * significant *p* value; n.s. not significant.

To prove that vitamin D increases *Fndc5* expression in myoblasts by acting through *Sirt1*, we used siRNA to knock down *Sirt1* expression in C2C12 myoblasts. qPCR analysis showed that 48 h of treatment with vitamin D significantly upregulated *Fndc5* mRNA expression in non-targeting siRNA control cells (scramble-silenced; scr siRNA) (*p* = 0.001); however, this effect was completely blunted in *Sirt1* silenced cells (*Sirt1* siRNA) (Figure 4A). Moreover, *Fndc5* mRNA expression was significantly reduced in the untreated myoblasts silenced for *Sirt1* compared with the untreated control cells (*p* = 0.048), as well as in vitamin-D-treated myoblasts silenced for *Sirt1* compared with vitamin-D-treated control cells (*p* = 0.016) (Figure 4A). At the same time point (48 h), *Sirt1* mRNA expression was strongly downregulated and barely detectable in *Sirt1*-silenced myoblasts (Figure 4B) (*p* = 0.0002), suggesting that the lack of effect of vitamin D on the upregulation of *Fndc5* was probably caused by the absence of *Sirt1*. Additionally, Figure 4C shows that in the absence of *Sirt1*, *PGC1α* mRNA expression was significantly reduced (*p* = 0.002).

## 3. Discussion

In the present study, we show that six-month treatment with vitamin D in PHPT with associated hypovitaminosis increased the irisin serum levels. Furthermore, our in vitro experiments confirm this effect by showing that stimulation with vitamin D in skeletal muscle cells enhanced the expression of *Fndc5*, the precursor of irisin. A relationship between vitamin D and irisin has been hypothesized in recent years, as both molecules are important regulators of the musculoskeletal system and energy homeostasis. However, to the best of our knowledge, this is the first study evaluating the effect of vitamin D treatment on irisin serum levels in patients with PHPT. The possible vitamin-D-dependent increase in irisin levels is supported by other recent preclinical studies. It had been observed that rats with hypovitaminosis D were hypoirisinemic [20], and vitamin D supplementation upregulated the *Fndc5* gene expression in the skeletal muscle of a diabetic rat model [21]. Previous studies have brought to light an existing negative relationship between irisin and vitamin D. We previously observed a negative association between irisin and vitamin D in a cohort of CMT patients [7], and in children with type 1 diabetes mellitus [8]. Unlike these previous studies, this cohort of PHPT patients was treated with cholecalciferol, allowing us to assess the impact of vitamin D on serum irisin levels.

Despite abundant evidence on the prevalence of hypovitaminosis D in patients with PHPT, some uncertainty prevailed regarding whether hypovitaminosis D should be treated in these patients, particularly because the resulting increase in vitamin D concentration might have a negative feedback on PTH production and secretion. However, our results show that PTH circulating levels after 6 months of vitamin D treatment were slightly reduced compared to baseline. In agreement with this result, a meta-analysis of 10 studies comprising 340 patients showed that vitamin D supplementation in patients with PHPT significantly reduced PTH values without causing hypercalcemia and hypercalciuria, and another study demonstrated that normalization of vitamin D nutritional status resulted in significant reductions in PTH levels without any safety concerns [16]. However, a limitation of this study is the small number of patients analyzed. Future studies involving a larger number of patients with PTPH, as well as a longer duration of treatment, will be needed to demonstrate that vitamin D treatment may be a therapeutic application for PHPT, or that it may result in an increase in circulating serum irisin.

Our in vitro results show that 48-h stimulation of vitamin D increased Fndc5 expression in myoblasts and irisin secretion in their cell culture media, while 8-h stimulation upregulated Sirt1 and PGC1α expression in these cells. By silencing the expression of *Sirt1* in myoblasts using RNA interference, we investigated its putative role in the vitamin D/Fndc5 pathway, demonstrating that vitamin D increases the expression of the irisin precursor in muscle cells only in the presence of an intact expression of *Sirt1*.

As previously demonstrated, vitamin D treatment promoted Sirt1 and AMPK activation in skeletal muscle cells [22]. Sirt1 is a nicotinamide adenine dinucleotide (NAD)-dependent protein deacetylase [23] that plays an essential role in AMPK activation [24]. As major regulators of muscle fiber oxidative capacity and mitochondrial biogenesis, AMPK and Sirt1 influence Pgc1α activation and transcription [18,19].

Pgc1α is a co-transcriptional regulator facilitating multiple transcription factors to regulate a complex network of genes [25], and it is involved in both the control of the tissue mitochondrial content and the program leading to brown adipose tissue (BAT) formation [26]. Consistently, it has been shown that the activation of Pcg1α increases the expression of the irisin precursor *Fndc5* [2].

Currently, there is limited and conflicting evidence on the effects of vitamin D on *Pcg1α* activation, and further studies will be needed to understand their relationship and whether this interaction is influenced by the tissue-dependent expression of vitamin D receptors (VDRs). However, given the critical role of *Fndc5* and its transcriptional activator, *Pcg1a*, in muscle mitochondrial changes induced by physical activity, the results of the present study, showing that vitamin D positively affects their expression in skeletal muscle tissue, add a piece of knowledge to the study of the mechanisms involved in this pathway.

**Figure 5:**
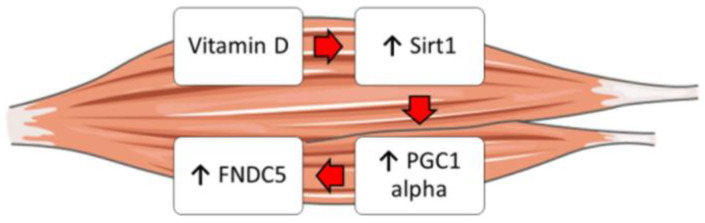
Schematic representation showing the possible effect of vitamin D on the Sirt1 > Pcg1a > Fndc5 pathway in skeletal muscle cells.

Moreover, it is well known that vitamin D enhances myogenic differentiation [27]. Irisin also has myogenic effects, promoting hypertrophy through activation of satellite cells and increasing protein synthesis [28]. Overall, our findings might explain these overlapping effects and suggest that vitamin D could increase Fndc5/irisin synthesis by enhancing the expression of Sirt1 and Pgc1a in skeletal muscle cells (Figure 5). Furthermore, these studies might support the fascinating idea that vitamin D possibly acts through irisin to promote myoblast differentiation and function.

## 4. Materials and Methods

### 4.1. Study Population

This study is a secondary analysis of a prospective, multicenter study performed in 29 Italian centers for endocrine diseases. Caucasian postmenopausal women with newly diagnosed PHPT were enrolled. The main exclusion criteria were taking anti-osteoporosis therapy, and, because renal dysfunction is also known to affect irisin levels, subjects were also excluded if they had renal failure with a glomerular filtration rate < 30 mL/min. Upon inclusion, weight and height were measured for the body mass index (BMI) calculation. These patients were treated with Dibase (2000 U/day) at the diagnosis of PHPT until follow-up after 6 months.

### 4.2. Biochemical Measurement

Blood samples were taken between 08:00 and 10:00 a.m. for measurement of the serum calcium, 25(OH)-vitamin D, parathyroid hormone (PTH), and irisin in the fasting state. Except for Ca^2+^, whose levels were measured immediately after collection (within 2 h of sampling, while the room temperature was maintained at 4 °C), the blood samples were aliquoted and stored at −80 °C. Irisin serum concentrations were detected using a competitive enzyme-linked immunosorbent assay (ELISA) kit (AdipoGen, Liestal, Switzerland) with an intra-assay coefficient of variation ≤ 6.9%. This ELISA kit allows for the largest range of measurement (0.001–5 µg/mL) and is the most sensitive (0.001 µg/mL). We followed the manufacturer’s instructions for all of the analyses. The colorimetric reaction was measured using a spectrophotometer (Eon, BioTek, Winooski, VT, USA) at the end of the assay. The serum 25(OH)-vitamin D concentration was measured using a commercial RIA (Diasorin, Stillwater, MN, USA), with 1.5 ng/mL sensitivity, and 7.2% intra- and <12% inter-assay CVs. Serum PTH levels were measured using an immunochemiluminescent assay (Liaison 1–84 PTH, Diasorin, Stillwater, MN, USA), with 2 pg/mL sensitivity, as well as 5.9% intra- and 8.3% inter-assay CVs.

### 4.3. Cell Culture

Mouse myoblast C2C12 cells were used for in vitro experiments in this study. Myoblast were plated at 10^3^ cells/cm^2^ and cultured in Minimum Essential Medium Eagle–Alpha Modification (α-MEM) (Gibco; Thermo-Fisher, Waltham, MA, USA) with 10% fetal bovine serum (Gibco; Thermo-Fisher, Waltham, MA, USA) until they reached confluence in a humidified atmosphere (37 °C, 5% CO_2_) (Hera cell 150; ThermoFisher, Waltham, MA, USA). For knocking down Sirt1, C2C12 myoblasts were transfected with Sirt1 siRNA (80 nM) or Scramble (scr) using Lipofectamine RNAiMAX Reagent (Invitrogen, ThermoFisher, Waltham, MA, USA) and then treated with 10^−8^ M of vitamin D for 48 h.

The concentration of irisin in the cell culture media was measured using a competitive ELISA kit (Phoenix Pharmaceuticals, Burlingame, CA, USA, Cat. No. EK-067-29), which allowed for the detection of a very low concentration of protein (ng/mL). This kit had a sensitivity of 1.29 ng/mL, linear range 1.29–27.5 ng/mL, intra-assay coefficient of variation (CV) of <10%, and inter-assay CV of < 15%.

### 4.4. Vitamin D Treatment

The cells were treated with 10^−8^ M of the biologically active form of vitamin D, 1α,25-dihydroxyvitamin D3 (1,25(OH)2D3) (Sigma-Aldrich, St. Louis, MO, USA) or with control solution (DMSO) for 8 h, 48 h, and 72 h. The dose–response experiment was performed by treating the myoblasts with a concentration between 10^−7^ M to 10^−9^ M of vitamin D.

### 4.5. Real Time PCR

RNA from the cells was extracted using the RNeasy Mini Kit (Qiagen, Hilden, Germany) using spin columns following the manufacturer’s instructions. We performed reverse transcription by iScript Reverse Transcription Supermix (Bio-Rad, Hercules, CA, USA). As a thermal cycler, we used MyCycler (Bio-Rad, Hercules, CA, USA) according to the manufacturer’s instructions. We completed Real Time PCR using the SsoFast EvaGreen Supermix (Bio-Rad, Hercules, CA, USA) on a CFX96 real-time system (Bio-Rad, Hercules, CA, USA) for 40 cycles (denaturation, 95 °C for 5 s; annealing/extension, 60 °C for 10 s) after an initial 30-s step for enzyme activation at 95 °C. We used Primer-BLAST to draw primers (https://www.ncbi.nlm.nih.gov/tools/primer-blast/ accessed on 15 February 2023). *Gapdh* was chosen as a housekeeping gene because it was stably expressed in all of the samples. The primer sequences were as follows: *Gapdh* (S-acaccagtagactccacgaca, AS-acggcaaattcaacggcacag); *Fndc5* (S-ctcgttgtccttgatgata, AS-attgttgtggtcctcttc); *PGC1*α (S-ccctgccattgttaagacc, AS-tgctgctgttcctgtttttc), and *Sirt-1* (S-tcggctaccgaggtccata, AS-cgctttggtggttctgaaagg).

### 4.6. Western Blot

Here, 20 µg of protein from the myoblast cell cultures were solubilized with a lysis buffer (50 mM Tris (Tris(hydroxymethyl)aminomethane)-HCl (pH 8.0), 150 mM HCl, 5 mM ethylenediaminetetraacetic acid, 1% NP40 and 1 mM phenylmethyl sulfonyl fluoride). The protein concentration was measured using a DC™ Protein Assay (Bio-Rad Laboratories, Hercules, CA, USA). The cell lysates were subjected to SDS-polyacrylamide gel electrophoresis (SDS-PAGE) and then transferred to nitrocellulose membranes (Sigma-Aldrich, St. Louis, MO, USA). The blots were incubated overnight at 4 °C using primary antibody anti-Fndc5, anti-Sirt1, anti-Pgc1α, and β-Actin (Abcam, Cambridge, UK). Subsequently, the membranes were incubated for one hour at room temperature with IRDye-labeled secondary antibodies (680/800 CW) (LI-COR Corp., Lincoln, NE, USA). The Odyssey infrared imaging system (LI-COR Corp., Lincoln, NE, USA) was utilized for immunodetection. All data were normalized to loading controls.

### 4.7. Statistical Analysis

Analysis of the sample distribution was performed by D’Agostino and Pearson normality test. Parameters with a normal distribution were expressed as mean ± standard deviation (SD), while parameters with a non-normal distribution were expressed as median and interquartile range (IQR). By using GraphPad Prism (GraphPad Software, Inc., La Jolla, CA, USA), we performed unpaired *t*-test or one-way analysis of variance (ANOVA) for values that passed the normality test, whereas for non-normal distributed values we performed Mann–Whitney test or the Kruskal–Wallis test. The results were considered statistically significant for *p* < 0.05. ImageJ was used to process the images.

## Figures and Tables

**Table 1:** Mean (±SD) or median (25%; 75%) for all variables.

	All Patients (*n* = 16)
Age (years)	58.69 ± 7.54
BMI (kg/m^2^)	26.05 (24.13–30.95)
Ca (mg/dL)	9.76 ± 0.55
PTH (pg/mL)	116 (76.75–134.5)
25(OH)-Vit D (ng/mL)	14.82 ± 8.66
irisin (mg/mL)	3.85 ± 1.44

## Data Availability

The data that support the findings of this study are available upon request from the corresponding author.
